# Dermatomyositis Leading to Necrotizing Vasculitis: A Perfect Response to Applied Therapy

**Published:** 2016-12

**Authors:** Mahmood Akbaryan, Farideh Darabi, Zahra Soltani

**Affiliations:** 1 Professor of Medicine, Rheumathology Research Centre, Tehran University of Medical Sciences, Tehran, Iran;; 2 Fellowship of Rheumatology, Rheumatologic Research Center, Tehran University of Medical Science, Tehran, Iran

**Keywords:** Dermatomyositis, Necrotizing, Vasculitis, Muscle, Skin

## Abstract

Dermatomyositis is an idiopathic inflammatory myopathy that cause skin and muscle complications. The ethiology is not understood well yet. Released cytokines including interferon and interleukins are suggested to make inflammatory responses in the skin or muscle. Muscle weakness and skin lesions including heliotrope rash, shawl sign and Gottron’s papules are the most common symptoms. A biopsy (muscle or skin) is always the most reliable method for diagnosis. Corticosteroids in association with immunosuppressive agents are used as standard treatment. The patient was a 30 years old woman who got involved with dermatomyositis for 10 years. She has been under therapy with Methotrexate, Prednisolon and Azathioprine until she came to us suffering from progressive skin lesions. Experiments and examinations were normal except the lesions and detected lipoatrophy. Because of immune cells infiltration and observations necrotizing vasculitis was diagnosed. After three month of high dose prednisolon and intravenous cyclophosphamide therapy the lesions vanished remarkable. True and immediate diagnosis gives physicians the chance not only to assess the best treatment but have adequate time to apply the procedure. However shortening the therapy and diminishing morbidity of the disease need more investigations and efforts.

## INTRODUCTION

Dermatomyositis as a connective tissue disease syndrome is an idiopathic inflammatory myopathy that affects cutaneous characteristics and striated muscles. The disorder is rare, with a prevalence of one to 9.63 cases per million in adults and one to 3.2 cases per million in children (1). It indicates solitarily or could be associated with other disorders of connective tissue (overlap syndrome) and also malignancy (2).

The etiology of dermatomyositis is not perfectly recognized yet however the most agreeable cause is reported due to genetics and particular genes over expression (e.g. type 1 interferon–inducible transcripts) leading to autoimmune responses that cloud be triggered by endotheliotropic viruses and underlying neoplasia. Uncommon drugs, namely the lipid-lowering agents, also have been reported to implicate dermatomyositis (3). Although the disease can onset at any age, 40 years is the most reported average age at the diagnosis, and women are affected almost three times more than men. Dermatomyositis can indicate in children too which is called juvenile dermatomyositis. It could onset at the average age of 5 to 14. These subgroups have a better prognosis than adults (4, 5).

The pathologic features of dermatomyositis including autoantibody production, endothelial damage, dysregulation of both complement and mannose binding lectin, and impaired clearance are active systemically (6). When immune complexes bind to endothelium cells complement system activates and cell lysis occurs that leads to cells necrosis, and capillaries in the muscle reduce in number. These complement components could get visible immunohistochemically on muscle capillaries (7).

Released cytokines especially type I interferon (TNF-α) and interleukin-1 (IL-1) trigger downstream cascades and cause inflammatory responses in the skin or muscle that lead to dermatomyositis indications (8).

Symptoms usually manifest in to main tissues, skin and muscle. Skin lesions as the most common sign of the disorder including heliotrope rash usually around the eyes, shawl sign commonly over shoulders, arms and upper back, Gottron’s papules often appear over the elbow, and knee joints and in V-shaped signs distribute over the anterior neck and chest. Presentation of many systemic symptoms also may occur in patients with dermatomyositis of which proximal muscle weakness is more likely. However approximately 2 to 11 percent of amyopathic patients with dermatomyositis do not indicate evidence of muscle involvement. These patients suffer from lethargy, pruritus, fatigue, photosensitivity or arthralgias more likely (9). These lesional propagations have been referred to antibodies of microvasculature endothelium of the skin, muscle and lung (3). Other possible symptoms include respiratory muscle weakness, visual changes, abdominal pain and internal malignancy. Among them malignancy needs more observance and care (2). Despite ovarian and gastric cancer and lymphoma as the most commonly reported malignancies, lung, male genital organ, nonmelanoma skin, Kaposi’s sarcoma, mycosis fungoides and melanoma are other types of reported malignancies in patient with dermatomyositis (3, 9).

Electromyography is widely used to diagnosis of dermatomyositis other efficient methods also have been taken advantage such as whole-body MRI that has proved as an alternative assessment to state of the disease truly (4) however a biopsy (muscle or skin) is always the most gives the most correct and reliable diagnosis (7).

Thanks to modern therapy the mortality has reduced significantly from about 50 percent to less than 10 percent. The most suggested treatment for idiopathic inflammatory myositis is applying Corticosteroids and the treatment is usually initiated with pulsed intravenous glucocorticosteroids (250–1000 mg Prednisolone per day). Oral treatment is also beneficial that consists of prednisone 1 mg/kg/day (7).

Sometimes because of corticosteroid resistance or tolerance additional immunosuppressive agents such as cyclophosphamide are inevitable for instance Azathioprine is used in chronic inflammatory diseases and Methotrexate suppresses inflammation and improves function (5).

*Necrotizing vasculitis:* it is an inflammatory necrotizing disease of the blood vessel walls in association with neutrophil infiltration. It affects small to medium-sized vessels (e.g. capillaries, venules, arterioles, and arteries) (7).

It commonly occurs secondary to autoimmune diseases, including connective tissue diseases. Pathogenic mechanisms including cell-mediated inflammation, immune complex-mediated inflammation and autoantibody-mediated inflammation. Evidences strongly suggest that immune complexes at the site of vessel wall deposition response to chemotactic factors elaboration of the complement cascade released of deposition of. IgG or IgM are the most released antibodies (IgA rarely releases) (10).

## CASE PRESENTATION

The case was a 30 years old female with a 10 years background of dermatomyositis (DM). She complained about skin lesions. In her medical history characteristics have been recorded as follows: muscular weakness, high level of muscular enzymes, inflammatory myopathy indicated by electromyography (EMG) and heliotrope and Gottron’s rash. She has been under the treatment by Prednisolone, Methotrexate and Azathioprine since the disease commenced. She was examined for malignancies at the disease onset and no particular subject was found.

**Figure 1:**
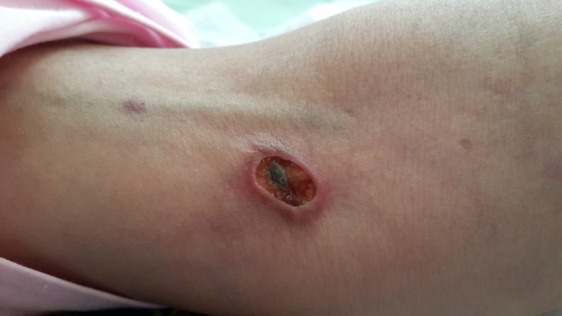
Skin ulcer.

The patient has been suffering from disseminated skin lesions with priority on organs since about a year ago that occurred gradually. Painful erythematous lesions were indicated as papules at the beginning which started to get bigger gradually and sometimes associated with secretions (Figure 1).

Until a year and half ago, prior to skin lesions, the patient was taken following medicines: Metotrexate in 5 mg, Prednisolon in 10 mg and Azathioprine in 50 mg. Since the mentioned lesions indicated the medicines were changed by the physician as follows: Metotrexate in 5 mg > 25 mg/w, Prednisolon in 10 mg > 30 mg/d and Azathioprine in 50 mg > 150 mg/d. Despite of medicine dose increscent lesions did not heal. When the patient came to our center she didn’t complained about muscular weakness. Through the examinations; Head and neck: not pale conjunctiva, icterus in conjunctiva and mucus was not detected. Heart: S1 and S2 were heard normally, murmur was not heard. Lung: clear. Abdomen: soft, no tenderness and organomegaly. Joints: normal. Mussels: had normal power. Skin: 20 lesions detected, most of which on organs and a few on trunk and hips, no Calcinosis. Organs: lipoatrophy on thigh, arm and abdomen.

**Figure 2:**
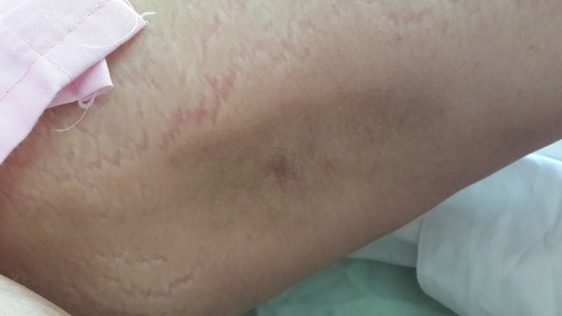
Lipoatrophy on tight.

Results of experiments taken by this center were as following: CBC: Normal, CPK: Normal, LDH: Normal, LFT: Normal, ESR =60, CRP =40, CXR: Normal, C-ANCA: -ve, P-ANCA: -ve, Anti Centromere: -ve, Anti Scl-70: -ve, Anti-Jo-1: -ve, Abdominal & pelvic CT scan:NL, Lung CT scan:NL, Mamography:NL, Limb radiography:no evidence of calcinosis (Figure 2).

Skin consultant proposed the probability of vasculitic rashes. Skin biopsy was applied and the treatment was changed in to pulse cyclophosphamide monthly and Prednisolon 1 mg/Kg.

**Figure 3:**
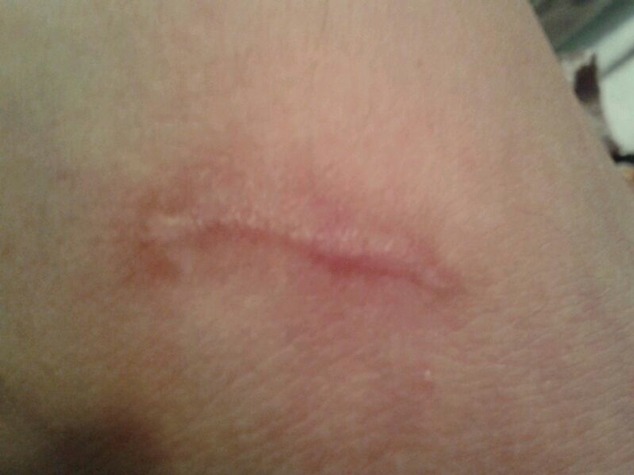
Skin ulcer 3month after treatment.

After a month lesions were healed slightly. After next 3 month lesions were healed remarkably (Figure 3) and there was not indication of fresh rash. Pathology reports were revealed as following: Infiltration of many neutrophil and a few eosinophyl were detected, Rbc extravasation, deposition of complement and IG were observed, transmural edema and necrosis were also seen. According to above the diagnosis was necrotizing vasculitis.

## DISCUSSION

Skin and muscle issues including rash as the most common skin indication that occurs early in 30 to 60 percent of patients and muscle weakness (up to 90%) has been widely reported as the most frequent diagnosis of dermatomyositis (2, 11).

Based on literature dermatomyositis is diagnosed by EMG and biopsies commonly as our case had been diagnosed through mentioned methods as well but due to a previous study sometimes it happens that dermatomyositis might not be found by standard methods. For these cases taking advantage of magnetic resonance imaging (MRI) has been reported to be a reliable method to diagnose the disorder (9).

Our patient was investigated carefully for malignancy at the time of diagnosis for three main reasons: 1- as a woman our case was exposed to malignancies more commonly (especially breasts and ovaries as there is a significant association with ovarian cancer in women with dermatomyositis), 2- skin changes are not different in patients with or without malignancy and 3- malignancies are the most important factors that could risk the survival and increase the mortality remarkably. Despite the gender, age is the most crucial risk factor to get involved with malignancies which during a study it was suggested a 6.5-fold increased risk of malignancy in patients diagnosed with dermatomyositis after 45 years of age (9).

The previous therapies has been applied based on literature and despite progressive erythematous lesions as the main reason the case came to us, all of experiments and examinations we have accomplished were normal but lipoatrophies and skin lesions. So the previous therapies could successfully affect the muscle weakness and decreased muscle enzymes level that could be a confirmation of prior studies (7).

As we report an association of dermatomyositis with lipoatrophy for our patient this relation is widely confirmed and has been indicated that dermatomyositis more than other autoimmune diseases may affect lipoatrophy in both duration (from 3 month to 11 years) and intense (partial or total). Age is also the only predictor for diagnosing the complications development, the younger patients were the more increased risk would have been (12, 13) and lipoatrophy has been infrequently reported in adult dermatomyositis (8).

While it is obvious that skin lesions are damages to keratinocytes and some observers have suggested that cytotoxic T cells are responsible for keratinocyte death in dermatomyositis, many images have been published of vacuolar keratinocyte injury lacking of any nearby immune cells. Despite this presence of cellular components of the immune system leading to tissue injury is commonly occurred and cytokines as released factors from intravascular wall cells are more likely to affect of attracting immune cells leading to tissue injury (14). Thus the presence of immune cells, T cells, B cells, macrophages and plasmacytoid dendritic cells are proved in perimysial and perivascular areas (7) and infiltration of some of mentioned cells in the inflammatory muscle biopsies have been confirmed in past studies (6). In following study occurrence of both complement deposition and immune cell infiltration was revealed as well and presence of cellular components of the immune system in the lesions was confirmed. These inflammatory responses may caused damages to small and medium vessels and led to necrotizing vasculitis as the diagnosis.

Among reported treatments as our application revealed, high dosage of intravenous Prednisolon injection accompanied by an immunosuppressive agent seems to be the most effective one that takes times which could be particular for each patient and variable from person to person (7).

## CONCLUSION

We reported a 30 years old female with a history of dermatomyositis who presented with necrotizing vasculitis and lipoathrophy 10 years after disease onset. This indicates the importance of attention to rare symptom in adult dermatomyositis like skin ulcer due to necrotizing vasculitis and lipoathrophy.skin ulcer and lipoathrophy are common symptomsss in juvenile dermatomyositis So that physicians would rather take the best treatment early. Muscle weakness and skin lesions are key factors to diagnose the disease and treatments are progressed adequately to minimize mortality so patients are very hopeful to survive. The remained concerns including time, patience and morbidity diminishing.
